# Forensic science capacity development: A case study of Timor-Leste

**DOI:** 10.1016/j.fsisyn.2024.100553

**Published:** 2024-08-31

**Authors:** Loene M. Howes, Roberta Julian, Renee Wilson, Maria C. dos Santos

**Affiliations:** a Tasmanian Institute of Law Enforcement Studies, School of Social Sciences, University of Tasmania, Private Bag 22, Hobart, Tasmania, 7001, Australia; b Timor Leste Police Development Program, Palm Business & Trade Centre, Block H, Rua Hudilaran (Banana Road), Surik Mas, Dili, Timor-Leste; c National Forensic Team, Polícia Nacional de Timor-Leste, Timor-Leste

**Keywords:** Sustainable Development Goals, Police development, Fingerprint examination, Global south, Timor-Leste

## Abstract

Forensic science is socially constructed within particular contexts, with notable challenges for countries of the Global South. This study explored forensic capacity development taking place under a bilateral agreement between the governments of Timor-Leste and Australia through the Timor-Leste Police Development Program. Data were collected through in-country site visits, observations, and interviews with key personnel from both countries. The findings indicate tangible developments, including the establishment of a forensic fingerprint laboratory, training in fingerprint expertise from crime scene to court, and engagement in innovative practices. These developments contribute to optimism amongst leaders and practitioners about the potential for forensic capacity. However, concerns exist about the precariousness of achievements, the need for continued training and development within and beyond the forensic team, untapped potential for inter-agency collaboration, and other human resource considerations. The findings suggest a need for organisational commitment and ongoing high-quality partnerships to maintain momentum and facilitate long-term sustainability.

## 1 Introduction

Forensic science is a multi-disciplinary field of endeavour with recent and rapid developments that offer exciting possibilities, such as field deployable instruments, which can be used by non-specialists at crime scenes and in the aftermath of disaster incidents [1]. Although such developments can become more affordable over time and at scale, the initial outlays and the complexity of validating new instruments, such as Rapid DNA, can be prohibitive for small laboratories, even in well-resourced countries of the Global North [2]. These kinds of issues are further compounded for forensic laboratories in developing countries and post-conflict states of the Global South [3], where many scientific and technological advancements are further from reach. Rather than aiming to replicate forensic science service provision in the Global North, achieving sustainable development in the Global South necessitates fit-for-purpose forensic science that addresses the strategic priorities in specific contexts [[4], [5], [6]]. The purpose of forensic capacity development is to contribute to improving criminal justice systems in line with United Nations Sustainable Development Goals (SDGs),^1^ including SDG 16 (peace, justice and strong institutions), SDG 10 (reduced inequalities within and between countries), and SDG 5 (gender equality) [4]. Reflecting SDG 17 (partnerships for the goals), working to achieve these long-term goals requires partnerships between countries.

This paper reports the findings of a study of forensic capacity development, undertaken as part of the Timor-Leste Police Development Program (TLPDP), which is supported by the Governments of Timor-Leste and Australia. The study adopts a critical forensic studies perspective, which recognises forensic science as a social process, influenced by particular economic, political, and cultural contexts [7]. This perspective also recognises that power relations and contested knowledge are inherent in justice processes. It assumes a social justice orientation, such that the goal of using forensic science is to improve the fairness of the system and enhance justice for all [7]. The paper proceeds by discussing forensic capacity development, introducing the context of Timor-Leste and the TLPDP, and outlining the data collection process. It then presents the findings on forensic capacity development in Timor-Leste and discusses implications. Overall, the findings suggest that the TLPDP, in partnership with the *Polícia Nacional de Timor-Leste* (PNTL), has made valuable inroads in forensic science by building the infrastructure of a forensic laboratory, strengthening skills in crime scene examination, and generating capacity in the fingerprint discipline from crime scene to court. However, for sustainable development, there is a need to nurture and expand this fledgeling capacity and maintain momentum through organisational commitment, strategic planning, and long-term support.

### 1.1 Forensic capacity development

In many countries of the Global South, there is an imperative to enhance justice in line with United Nations SDGs. Introducing forensic disciplines and improving the use of forensic science is part of this agenda. However, forensic science service provision poses a substantial resourcing challenge [3,6,8,9]. There is a vast array of forensic disciplines, each needing their own specialist knowledge and equipment. A range of related challenges exist, such as funding laboratory infrastructure, instruments, and safe storage (e.g., some chemicals need temperature-controlled environments, yet power outages can be common [4]). There is a need to attract, retain and develop specialist personnel, and provide necessary consumables, such as personal protective equipment and chemicals, generating challenges in meeting ongoing costs and often-disrupted supply chains [5,9]. Even if police investigators are trained, for example, to improve the response to sexual and gender-based violence, the system may lack a forensic laboratory or enough qualified judges to adjudicate cases [10,11]. Resourcing issues are often compounded by a lack of legislative and regulatory frameworks, and appropriate standard operating procedures, to guide and facilitate the use of forensic science [3,5]. Further, a lack of knowledge of forensic science among police and legal practitioners can mean that the value and relevance of forensic science evidence are not understood in comparison to other evidence types [3,5,8]. These intertwined issues make forensic capacity development a substantial challenge.

A key aim of forensic capacity development is to respond to human rights concerns. The intention is to improve the robustness of evidence to inform police and legal decision making, to provide greater trustworthiness of the system, to help victims, offenders, and the community, particularly when police corruption or trustworthiness has been an issue [3]. In developing and post-conflict countries, addressing sexual and gender-based violence is often a national priority that forensic evidence can help address [[11], [12], [13], [14]]. However, donor countries’ priorities and international agendas also influence the nature and scope of policing assistance [15]. For example, it can be beneficial for host countries to support regional responses to transnational crime, such as enhanced drug detection capability (e.g., Refs. [16,17]). A further purpose for developing forensic capacity is for effective disaster responses – especially given the increased risks of natural disasters associated with climate change and the larger numbers of deaths in countries with lower capacity for investment in preparation and mitigation strategies [18]. Local capacity is needed, and arguably, the best way to build forensic capacity for disaster responses is to improve the forensic capacities of the system generally [19].

Important changes have occurred in the rationale and ethos for development programs. For example, the United Nations Development Programme (UNDP) notes that while the focus was once on *development aid*, then *technical assistance*, it subsequently shifted towards *technical cooperation*, before making a more significant shift to *capacity development* [20]. This shift reflects increased understanding of local agency as a crucial element of sustainable development. The UNDP defines capacity development as “the process through which individuals, organisations and societies obtain, strengthen and maintain the capabilities to set and achieve their own development objectives over time” ([20], p. 4). Internationally, police capacity development programs in developing and post-conflict countries aim to build, rebuild, or reform police organisations and justice systems to provide increased quality of – and access to – justice [17]. These programs often focus on broad policing objectives, such as leadership and general training, gender-responsive policing, and responding to specific crime threats, such as terrorism or cybercrime. When they focus on expanding investigative capacity, programs sometimes include a small component of forensic science through the provision of infrastructure and training [[21], [22], [23], [24], [25]].

Forensic science capacity development occurs through intergovernmental organisations – such as the UNDP, the United Nations Office on Drugs and Crime (UNODC), and Interpol, with experts sourced internationally (see e.g., Refs. [3,8,9]). It is also provided by countries through governmental agencies with specialist forensic remits, such as the International Criminal Investigative Training Assistance Program (ICITAP) of the United States Department of Justice [[26], [27], [28]]. The Australian Government's support for police development programs in the Indo-Pacific region is implemented by the Australian Federal Police [[29], [30], [31]]. Additionally, bodies such as the International Committee of the Red Cross (ICRC) provide humanitarian forensic action, which includes forensic capacity development for local authorities [32]. Forensic medicine and forensic science discipline-specific associations also contribute to forensic capacity development (e.g., Ref. [33]). Further, research can help develop innovative approaches to address issues of the sustainability of forensic science in the Global South. For example, researchers have considered locally available alternatives to chemicals for fingerprint examination in Brazil and Seychelles using a “frugal forensic approach”, reducing the costs of purchasing and storing chemicals [4,34]. Researchers have also explored the use of self-administered intimate DNA-swabs for sexual violence cases in low-resource environments [6].

Despite the range of forensic capacity development initiatives that occur, as a relatively small and specialist area internationally, they are not always well understood [16,23]. Evaluations of police capacity development programs typically report on the forensic science component only briefly (e.g., Refs. [[23], [24], [25]]), and research exploring specific forensic science capacity development initiatives is limited. However, program evaluations of police capacity development programs have reached similar conclusions, which are relevant for forensic capacity development. Essentially, these evaluations highlight that for effective and sustainable change, programs must be planned and developed collaboratively to meet the needs and visions of the host country. They must consider the justice system/s and processes holistically to ensure their ongoing cohesion. They must work with other donor partners and agencies in related areas to avoid duplication of efforts and ensure compatibility of goals (see e.g., Refs. [17,35,36]).

### 1.2 Context of Timor-Leste

Timor-Leste is located on the eastern side of the island of Timor, a beautiful mountainous island. It consists of thirteen municipalities, one of which, *Oecussi*, is a coastal exclave on the western side of the island, surrounded by Indonesian land. The country also has two smaller islands, the inhabited island of *Ataúro* off the coast of the capital of Dili, and the more remote uninhabited island of *Jaco*. Of the total population of 1.34 million, 65 % are under 30 years of age. Just 30 % of the population reside in urban areas, with 70 % based in rural areas [37]. Timor-Leste is classified by the United Nations both as a least developed country and a small island developing state [38], with a notable reliance on oil and gas revenue [39]. Timor-Leste's political engagement and optimistic international outlook is reflected in its role as headquarters of the g7+ (a group of conflict-affected states advocating for peace and development partnerships), its efforts to join Association of South-East Asian Nations (ASEAN), and its membership of the Community of Portuguese Language Countries. Present circumstances reflect how Timor-Leste has emerged from the struggle for independence.

Timor-Leste gained independence in 2002, after a prolonged period of around four-and-a-half centuries of colonisation by Portugal, interrupted by over 3 years of occupation by Japan during World War II, and followed by a 24-year occupation by Indonesia. The occupation and lead-up to independence were characterised by widespread conflict, sexual violence and loss of life, as well as the decimation of infrastructure [40]. The international community responded through five separate United Nations missions and two Australian-led multi-national stabilisation missions. Both stabilisation missions aimed at restoring security; the first in 1999 after the outcome of the referendum favoured independence, and the second in 2006 after riots arising from conflict between the new military and police institutions [41]. The UN missions began with assistance for the 1999 referendum, followed by the provision of transitional administration and support to establish institutions, such as the police, until Timor-Leste formally gained independence in 2002. Subsequent UN missions continued to support the development of institutions, including the police. Although UN missions were scaled down prior to the 2006 riots, they were scaled up in the aftermath. The International Stabilisation Force withdrew in November 2012 and the United Nations Integrated Mission in Timor-Leste concluded in December 2012 [41]. Since then, various countries have continued to provide bilateral support, and a host of UN agencies have maintained a presence in Timor-Leste alongside local civil society organisations [42,43].

The history of Timor-Leste has resulted in linguistic complexity with Tetum and Portuguese as official languages and Indonesian (*Bahasa Indonesia*) and English as working languages, alongside about 30 local languages [44]. Various countries offer educational scholarships, including Australia (where education is in English), Brazil (where education is in Portuguese), and South Korea (where education is in English for foreign students).

#### 1.2.1 Timor-Leste Police Development Program

A bilateral agreement exists between the Governments of Timor-Leste and Australia for the provision of various assistance under the umbrella of Australian Government's Department of Foreign Affairs and Trade (DFAT). Having commenced operation in 2004 [45], the TLPDP has been part of the suite of support provided by theAustralian Government for over 20 years. It is referred to in Tetum as *Serbisu Hamutuk* (“Working Together”). The fourth iteration of the program, from 2018 to 2022 (later extended to 2023) focused on three core components: (1) sustainability, self-improvement and leadership; (2) operational effectiveness; and (3) responsive policing services. It included a focus on improving the detection, response, and investigation of crime in general, and of transnational and organised crime specifically [46].

A forensic advisor commenced in January 2020 to conduct a scoping project [47]. The TLPDP team is led by a senior responsible officer and includes advisors in gender; human resources; maritime; monitoring, evaluation, and learning (a role that was included later); training; transnational crime; and security. Additional staff support the project, including locally engaged staff in Timor-Leste who provide language assistance, and AFP members based in Australia. Advisors receive some discretionary funds to support strategic objectives. An evaluation of the TLPDP [25] reported that the PNTL's new Transnational Crime Centre (funded by the TLPDP) had a space for a forensic laboratory with the purpose of supporting fingerprint examination through physical and chemical detection, optical examination, and digital photographic capabilities [25]. As some PNTL members had previously trained with Australian police officers of the Northern Territory Police Force (NTPF) in 2018 and a delegation had undertaken a study visit in 2019, the evaluation suggested that due to the proximity, cost, and shared rural/remote focus, it may be valuable to explore further links between the PNTL and NTPF [25].

### 1.3 The present study

As outlined, forensic capacity development is challenging for a range of reasons. However, if effective, it has the potential to impact positively the professionalisation of the police through increased effectiveness of investigations, prosecutions, and justice outcomes for the community. Although researchers have examined police capacity development in general, to date limited research has explored forensic-specific capacity development and the partnerships involved. Timor-Leste was selected as a site for the pilot study due to Australia's relatively long involvement in peace and security in Timor-Leste [45], and the recent forensic capacity development component of the TLPDP [25]. The present study aimed to explore forensic capacity development in Timor-Leste from a critical forensic studies perspective to better understand how forensic science is socially constructed in the specific context.

## 2 Method

### 2.1 Sample and participants

We obtained ethical approval for the study from the University of Tasmania Human Research Ethics Committee (H0029181). We obtained organisational approvals from the AFP (International Forensic Engagement and TLPDP) and the PNTL (National Forensic Team). Due to the proximity of the NTPF's Forensic Science Branch in Darwin to Dili, and its role as a destination on a PNTL study tour in 2019, we also obtained approval for research from the NTPF.^2^

In-country research in Timor-Leste involved one week of observations and site visits to the TLPDP facilities and PNTL forensic laboratory in Dili in Timor-Leste. It also included a total of eight formal interviews, two with locally engaged staff of the TLPDP (1 female and 1 male member), who were assigned to support forensic capacity development, and six with members of the National Forensic Team of the PNTL (2 female and 4 male members), from a total of seven members at the time. Some of the locally engaged TLPDP members and National Forensic Team members had tertiary education (not necessarily in science), sometimes undertaken overseas. Members of the National Forensic Team had differing levels of experience in policing and forensic science, ranging from those who had been transferred recently to the section to those who had had formative experiences accompanying UN forensic experts during the early days of the PNTL and the country's independence.

Informal interviews were undertaken with five AFP members deployed to the TLPDP (2 female and 3 male members) and informal discussions occurred with others. Australian participants had experience not only in Australian policing but also in previous overseas deployments, including some who had previously been deployed to Timor-Leste in other capacities. In Australia, the research included a site visit and tour of facilities of the Forensic Science Branch of the NTPF and informal interviews with two forensic scientists (2 female members), as well as informal discussions with several laboratory personnel. Leaders were included among each group of interview participants.

### 2.2 Procedure and interview guide

Project documents were translated into Tetum by a NAATI-recognised practising translator and checked for organisational nuances in Timor-Leste by a locally engaged language assistant of the TLPDP. These documents included the Participation and Consent Forms, a confidentiality agreement for interpreters, and the interview guide. Potential participants read the Participant Information and Consent Forms in Tetum or English, depending on their preference, and had the opportunity to ask questions about the project. They provided written or verbal consent to participate in interviews. For interpreter-assisted interviews, the process of working with the interpreter was explained. All interviews with local participants were audio-recorded and transcribed.

The interview guide asked participants about their role in their organisation, their involvement in the forensic science capacity development project, the benefits and challenges of the project including any examples they wished to provide, and what they would like to see in next steps for the future. The informal interviews with AFP members deployed to the TLPDP, and with personnel at the Northern Territory Forensic Science Branch, were primarily to help us to understand the contexts of the work from participants’ perspectives. We made notes during and after the discussions, with the opportunity for later follow-up questions and clarification via email.

### 2.3 Data analysis

Data for the study consisted of recorded interviews with members of the PNTL and locally engaged staff of the TLPDP, which were transcribed verbatim. For interpreted interviews, only the interpreter's renditions in English were transcribed. Additional data consisted of notes from informal interviews, written observations, and reflections on the visit to assist with contextual understanding. Interview data were analysed thematically, using the process outlined by Braun and Clarke [48]. This involved first listening closely to interviews several times while transcribing them. It then involved reading all contextualising notes. Next interview transcripts were read, and phrases, sentences, or paragraphs were coded based on the key points discussed. Codes were grouped to form tentative themes. Themes were then refined through subsequent reading and re-reading. Finally, quotes that best reflected the sentiments expressed by participants were selected to illustrate themes. To help maintain anonymity, participants are referred to by organisation and interview number.

## 3 Findings

Analysis of the data resulted in five key themes: (1) complexities of context; (2) achievements; (3) meaning of the work; (4) concerns; and (5) future vision. These themes are discussed in turn below, with related subthemes.

### 3.1 Complexities of context

Participants shared the desire that the complexity of the post-conflict environment and the unique local context be understood by the international community.

#### 3.1.1 Legacy of conflict

The legacy of occupation and conflict in Timor-Leste meant that bones – possibly human bones – were often found:

In [our section], we do the crime scene investigation, laboratory, sometimes anthropology – because in Timor we find many human remains – so it’s important to have this kind of anthropology examination – and also autopsy. We also assist the pathology to do the autopsy. And regarding crime scene investigation, we work 24 hours even though sometimes we feel overwhelmed with a lack of members because we have many roles to do. (PNTL, Interview 8)

Although the forensic team was small, their role was broad and there was not yet a full range of specialists to call upon for support.

#### 3.1.2 Legal system

The legacy of Portuguese influence includes an inquisitorial justice system. Despite the small population in Timor-Leste, this extends to having a separate agency, the Criminal Investigation Scientific Police (*Polícia Científica de Investigação Criminal*; PCIC) under the Ministry of Justice, while the PNTL is under the Ministry of Interior [25]:

After the establishment of PCIC, and because of … laws that exist, any major crime – like corruption, drug-related crime, homicide, human trafficking and smuggling – is allocated to the PCIC. And the police criminal investigation section, we investigate like simple crime, minor crime, and assault …. But sometimes it depends on the situation – if there is a crime and if police attend to it first, then police will investigate and PCIC will accompany and [the other way around]. (PNTL, Interview 3)

It was unclear which forensic disciplines were fully operational at PCIC, although participants mentioned ballistics and document examination, which were also noted on the Interpol's Timor-Leste webpage.^3^ At the time of our visit, there had been little collaboration in forensic science between PNTL and PCIC. Australian TLPDP members noted that there was political dialogue about merging PNTL and PCIC (Notes from TLPDP, Informal Interview); however, the timeframe for implementation was not known.

#### 3.1.3 Previous forensic capacity building

As a legacy of Portuguese colonisation, Indonesian occupation, and UN interventions, Timor-Leste is a multi-donor environment. In forensic science, donor engagement had included South Korea and China. Although PNTL members were interested in all training opportunities, for example, from Singapore and Australia, there had been past instances in which it was not clear how the training received would fit into the local context, or what a gifted item was and how it could be used:

The fume hood, that was the big one – the filter one, and it was very safe – but they didn’t use it because we don’t have the human resources – the people with knowledge or people that could study to broaden their knowledge – to use that equipment well. (TLPDP, Interview 7)

Forensic members of the PNTL had previously had access to training on how to collect reference fingerprints from people and finger marks from crime scenes. Previously, some longer-serving members had also been introduced to the Henry fingerprint classification system. However, storing paper records in flood-prone offices had proved disastrous, with many records destroyed over time (Notes from TLPDP, Informal Interview). In the Northern Territory context in Australia, forensic members noted that the Henry system was now used only in cases of historical crimes with the assistance of knowledgeable specialists (Notes from NTPF, Informal Interview).

Some forensic members had been involved with previous forensic-related training by the TLPDP:

Back in 2008–2010, there was Level 1, 2, and 3 Investigation Training … I was one of the participants, but in the forensic session, I was also asked to give some teaching about basic forensics – like how to use the … crime scene kit. On the forensic side, there was also training – the trainer was from Tonga, facilitated by the TLPDP – so we also had training on the crime scene photography, how to take a good photograph. There were also another two training [sessions] on how to collect fingerprints from the crime scene and how to compare them. I was not a participant in that training, but other colleagues were. (PNTL, Interview 3)

The training had included members from the regional municipalities. They had a workbook to complete and received a follow-up visit from the team:

Back then [2012], there was another investigation training team from TLPDP at the Police Training Centre and …. and at the end of this training, they provided a crime scene kit to participants where they can take it to the districts[/municipalities] … And then later we went to the districts[/municipalities] to check on the equipment of the crime scene kit and they tried to demonstrate back about how they’re using this equipment. One thing they demonstrated was the [use of] powder and brushes. (TLPDP, Interview 6)

Participants who had witnessed previous initiatives remembered them well. However, an organisational policy of moving police officers to new roles meant that most participating members were no longer in the forensic team, although it was unclear to what extent movement had occurred for those based in regional municipalities.

#### 3.1.4 Linguistic complexity

Although English is a working language, rather than an official language of Timor-Leste, English language skills were associated with opportunities within the PNTL [49] and within forensic science, such as access to online resources, conferences and training opportunities. For these reasons, English training was requested for the team and supported by the TLPDP.

Before, we were learning some Indonesian and Portuguese – but we are interested in English because with skills in English, we will really learn better forensic knowledge. (PNTL, Interview 1)

We try our best because we are a new country. If you choose not to be left behind, then we have to catch up with the things that are very important for us to develop, to evolve – it’s very important. Specifically in languages because if you don’t have this kind of skill you cannot catch up with anything. It sounds simple but its impacts are very big. (PNTL, Interview 8)

For language assistants supporting the forensic advisor to provide training, linguistic complexity was the norm, especially in specialist areas such as forensic science:

As an interpreter, we can use most of the [necessary languages] – we understand some different languages like Portuguese and *Bahasa* [Indonesian]. So, we try to act like a bridge for the knowledge and if we cannot find a word in Tetum, we use *Bahasa* so that people can understand. Sometimes it’s in the laws – like “fingerprint” is called “*impressão digital*” – and in our Tetum, we do use some words from Portuguese. And some parts of this [forensic] knowledge, the PNTL already have – so sometimes we also have to check – we might ask them, “What word would you normally use in your work?” (TLPDP, Interview 6)

To facilitate communication and cross-cultural learning, Australian members deployed to the TLPDP were able to take Tetum lessons over the lunch period once or twice weekly.

### 3.2 Achievements in forensic capacity development

Although some members of the PNTL had experienced previous forensic capacity development opportunities, recent initiatives represented a major leap.

#### 3.2.1 Establishing the laboratory

The previous TLPDP forensic advisor had been tasked with procuring equipment and instruments for the laboratory in the PNTL's Transnational Crime Centre. The third author initially took on a three-month deployment in 2022 to set up the forensic laboratory. At the time of our fieldwork, the forensic footprint in the Transnational Crime Centre included four rooms.

(1) the fingerprint laboratory, complete with a fume hood and superglue tanks,
(2) a photography room, with crime scene camera packs and various light sources,
(3) a room housing several computers, with the newly installed automated fingerprint identification system (AFIS), and
(4) a shared office space (which did not yet have internet connectivity) for the forensic advisor and forensic laboratory assistant.

#### 3.2.2 Training and development

Setting up the laboratory had taken less time than anticipated. A needs analysis had revealed that some members of the team had received limited crime scene photography training. Fingerprint examination is often done using photographs rather than tape lifts of finger marks, so photography skills are essential. Second, the focus had previously been on the collection of traces from crime scenes, but not their comparison and examination:

Before, we ended up with taking the evidence from the crime scene and it ended up in our office, even though we have some ninhydrin [chemical reagent for fingerprint detection on porous surfaces, such as paper and card] to use for the paper – but we didn’t have comparison. So, this project is very, very useful for our cases and motivates the members. (PNTL, Interview 8)

Training commenced and a longer-term forensic advisory deployment followed, with the third author the successful applicant. Training in fingerprint examination introduced the full process of detection, collection, analysis, comparison, evaluation, verification, and reporting.

What I’m participating in at this time, with [the forensic advisor] teaching us about the fingerprint until using the chemicals, I try my best to master this knowledge, and I try my best how to use the equipment here and to learn with [the laboratory technician] how to mix chemicals. (PNTL, Interview 3)

And the difference that I saw with them is that they catch up differently in the area. Some are very good in photography, even good in digital camera – they can configurate it very well. Some … have a very keen eye, so they are very good in fingerprints …. They saw right away differentiated characteristics … they learn the process and they can say, “Oh this one right away, left hand, right hand”. And even though we have automatically searching software, [the forensic advisor] said, “I want you guys to do it manually”. And I remember 40 fingerprints – they nailed it! (TLPDP, Interview 7)

A new role was also introduced:

There is a big change now to have a member who stays and prepares and looks after the equipment, prepares the crime scene kit to support the members who attend the crime scene. (PNTL, Interview 3)

Training extended to other friction ridge skin such as footprints and palm prints, and to other pattern evidence such as tyres and footwear. Notably, the examination of shoemarks was introduced only to assist investigators with actionable intelligence rather than to establish a footwear database, given a vast array of footwear imports from Indonesia.

#### 3.2.3 Building capacity in municipalities

A plan was in place to train two investigators from each municipality in fingerprint collection. At the time of our visit, one investigator from each of four municipalities had received three weeks of training and had their crime scene kits updated. The training included one week of basic photography, one week of crime scene processing and laboratory processing, and one week of scenario training to put it all together. The trained examiners were from neighbouring municipalities to help establish local professional networks. The National Forensic Team noticed benefits:

Now they can process a crime scene or a fingerprint without the assistance of the National Forensic Team. (PNTL, Interview 2)

Before, even though [the municipalities] had the 10-print form/template for fingerprints, they may not take them. After they attend this training, they can go back and take these fingerprints and submit to National here for doing the comparison – so that’s the benefit. (PNTL, Interview 3)

A new role was also introduced in municipalities:

What we are focusing [on] is.… having someone to take care of the equipment – a focal point – because the districts[/municipalities] have officers that are already good in crime scene examination. (PNTL, Interview 8)

#### 3.2.4 Forensic anthropology

Due to the forensic advisor's educational background and experience in forensic anthropology, mentoring in this area was possible. Some participants noted that they had previously received some forensic anthropology training:

Talking about forensic anthropology, [a forensic anthropologist] from Australia provided some training in anthropology and so from that time we have continued to use the training. (PNTL, Interview 1)

Participants valued highly opportunities for mentoring and practice in forensic anthropology, including broader collaboration:

And we decided we would also like to have anthropology training, anthropology examination. We do it maybe three times at the laboratory and bring the doctors here to have the complete examination … some members already attended anthropology courses – outside courses – and even in Timor, but with the presence of [the forensic advisor], we do the practice. (PNTL, Interview 8)

### 3.3 Meaning of the work

Participants expressed pride in what had been achieved to date. Developing forensic capacity meant a great deal to all involved in the project.

#### 3.3.1 Opportunities for learning

Participants were self-motivated and enthusiastic about gaining new knowledge and skills:

I was really happy [to join this team] because investigation is really an area where people are so interested to come and work in this area, but no one gets to do it and it’s a place that we can learn a lot more knowledge – so [I’m] really interested! (PNTL, Interview 5)

This is like an exercise, so after [the class], I keep looking at it and keep practicing so when I come back to look at it, it’s quicker for me. For example, if the topic of the lesson was fingerprint [characteristics], afterwards, I looked at the internet and tried to understand more and look at the differences. (PNTL, Interview 4)

#### 3.3.2 Teamwork and culture of inquiry

Participants were encouraged by the forensic advisor to develop an inquiring mind, conducting experiments (e.g., about fingerprints on windows in different conditions) to learn more about the nuances of fingerprinting. They were also encouraged to work together and share knowledge:

What we have been learning here, we share among us. What I learn, I share with them, and what they learn, they share to me, so that when we go to the crime scene, [we know what to do]. (PNTL, Interview 5)

A particularly exciting development was innovative research on locally available plants and materials that could be used to make substitutes for expensive fingerprint equipment, chemicals or powders [50]. For example, local plants could be used to make fingerprint powder and plastic storage containers could be used to make portable superglue tanks.

For example, sustainability – that’s what we did here. Because like, our forensic chemicals, we only get supplied from Australia with an agreement or a licence. And for the local shop, they are not going to – it’s risky. So [the forensic advisor] made a bunch of research and I also thought it might be good to try. We have a lab, we have our basic chemical reagents, so we can use for testing! And she has broad networking and so it’s amazing because you share something all over the world! (TLPDP, Interview 7).

#### 3.3.3 Professionalisation of the PNTL

Participants noted that their involvement in forensic science could help the organisation:

We would not expect before to learn this. But now because we have this knowledge, we can communicate the knowledge to someone who is higher rank than us. Because talking about the science is not looking at the ranks. (PNTL, Interview 2)

The developments at the newly established laboratory were of great interest to the hierarchy:

The General Commander and the Second-in-Charge, they really support us. That’s why they agree the TLPDP to help us – working with the TLPDP. And they also visit the laboratory many times to find out what we are doing now. So, they always support it, including the Minister of Interior – very supportive – yes, he visited the laboratory and talking about this program, the next program, and our careers. (PNTL, Interview 8)

#### 3.3.4 Contributing to justice in Timor-Leste

Some participants expressed their personal passion and commitment by using their education, enthusiasm and hard work to contribute to a better future for Timor-Leste and its people.

This is my country, my institution. I love my institution so much. All of us can do something for this country, and institution. This is not our company, this is you know, working for the people, the people’s servants, so it’s very … important to do our job, to do the best. If you do the small things to be perfect, then the big things will be easy. (PNTL, Interview 8)

I feel like helping people, helping my country is one of my duties as a citizen. We are already independent for 20 years but there is no big difference in our country. In this crime unit, forensic, it’s the most important one, I think, to make this country’s stability – keep you safe – in security. Because every crime that happens needs evidence and before they didn’t have equipment to support investigators. (TLPDP, Interview 7)

Thus, the opportunity for forensic capacity development was deeply meaningful for participants.

### 3.4 Concerns

Despite the pride in achievements to date in forensic capacity development, there was recognition that it was very early days and there was a sense of urgency for progress to continue.

#### 3.4.1 Precariousness of achievements

Participants recognised that these achievements in forensic capacity development would not be possible without partnership:

So, it's like … like very important to have this backup otherwise we cannot achieve. Honestly – otherwise we cannot have this kind of you know projects. To have this laboratory within two years is big – and fingerprints are in every [type of] case. (PNTL, Interview 8)

Australian participants were particularly concerned about the precariousness of achievements. The time-limited nature of advisory roles, with the blend of short-term (e.g., 3-month) and longer-term (e.g., 2- or 3-year) deployments generated a certain amount of pressure for Australian and locally engaged staff across the TLPDP. Concerns about time running out and other commitments to family, meant it was not always feasible for advisors to extend their stay even if the option existed. Some advisors had not received in-person handovers themselves and despite well-considered program design, there was general concern about how much continuity could be achieved in the short (1-week) handover to the next advisor or indeed whether there would be a next advisor given shifting funding priorities (Notes from TLPDP, Informal Interviews).

Although the PNTL currently has limited casework for fingerprint examiners, there is the potential for dramatic increases as it becomes embedded into the policing landscape. This suggests a need to ensure sustainability once the workload is higher. Importantly, it takes many years of education and training, practice, and experience, as well as proficiency and certification testing for a fingerprint examiner to be certified as an expert in Australia. Accordingly, there is a need for ongoing support and access to training for the forensic team in Timor-Leste so that their skills in crime scene examination are nurtured and continue to develop (Notes from TLPDP, Informal Interviews).

Australian members of the TLPDP were also concerned that the program had been scaled down in recent years. From their perspective, there was a need for it to be ramped up again, partly because of a large port that was being built by a French company in a public-private partnership [51], which could be expected to have implications for illicit goods. There were already indications that transnational crime, such as trafficking in people and illicit goods, was a growing concern due to a porous border with Indonesia. Additionally, there were concerns about exploitation, facilitated by increasing use of mobile phones as internet connectivity improved (Notes from TLPDP, Informal Interviews). The team consisted of members with high-level skills, and a cohesive and supportive approach. However, opportunities for mentoring in certain areas, such as forensic anthropology, depended on the unique blend of expertise of the forensic advisor who was assigned for a given period. A larger team would allow for some flexibility to distribute the work to reduce single-point dependency in key areas, provide within-team mentoring, and better enable continuity and long-term success (Notes from TLPDP, Informal Interviews).

#### 3.4.2 Human resources considerations

The main concern expressed by participants was the practice of rotating staff within the PNTL:

There is a challenge affecting the PNTL internally, which is where the PNTL members who have been trained by TLPDP tend to be transferred to a different area after training …. An investigator might not be trained in this area and he or she gets transferred [into it]. (PNTL, Interview 2)

It's up to the Commander to assign us – and if the Commander says “you go” it is a command or order. (PNTL, Interview 4)

This concern had wide-ranging impacts on the sustainability of forensic capacity. It required communication with the hierarchy about solutions from a human resources perspective to develop role descriptions, promotional opportunities, and succession planning within the organisation to retain expertise:

The more we establish the sciences, we need more sub-team leaders, right? Because now we have fingerprint. Later on, we’ll have ballistic. And later on, we’ll have digital forensics. We have autopsy, we have anthropology …. And [for team leaders] they need rank, at least for sergeant – and they can teach, we can teach the new members. The Minister of Interior understands and emphasised it – you cannot transfer people – we have to focus on their career, their ranks so they can stay here. We had a workshop to explain the roles of the forensic and the mission, what we are doing now and what we plan (and to train the focal point throughout the districts/[municipalities]) and why the Commander should support them. (PNTL, Interview 8)

Locally engaged staff are embedded in the TLPDP and can hold a great deal of organisational memory. To assist with continuity and sustainability in forensic science capacity, there was also a strong case to justify ongoing roles for members with valuable scientific and technical skills, such as laboratory maintenance, preparation of chemicals, and support for casework, and for those with specialist scientific language skills [Notes from TLPDP, Informal Interviews). It would be valuable to consider how to build skills among other locally engaged staff to avoid single-point dependency.

#### 3.4.3 Cooperation and collaboration

There was a recognised need for training beyond the specialist team for the PNTL more broadly:

I’m hoping for more training for investigators …. If our colleagues don’t have the knowledge, then charging from the prosecutor may not happen, so that means there needs to be training for all police officers. (PNTL, Interview 1)

However, there were local delays in commitment to a training schedule for recruits’ and sergeants’ courses and in local action on curriculum development (Notes from TLPDP, Informal Interview).

While it was mentioned that good relationships existed with legal practitioners, formal discussions and training sessions with prosecutors, defence lawyers, and judges about new capabilities with fingerprint evidence had not yet taken place:

So could be a challenge if investigators or prosecutors say, “Okay, the evidence that you provided or collected, like witness statements or CCTV camera, or someone recorded so there is already evidence to satisfy.” …. Sometimes there is this with the witness statement. Because the police report it to prosecutors and then they can proceed to take it to court but they say: “We are happy with what you have provided to us and we don’t need to prove more with a fingerprint.” (TLPDP, Interview 6)

Participants were particularly concerned that others involved in the justice process may not value the additional information that fingerprint evidence could provide:

If there is a homicide in the house and there are only two persons and it goes to court, the court is asking the suspect. But maybe, through the forensic investigation, maybe [it becomes apparent that] there was another person in the house. But because they already confess at court, the forensic evidence might be just kept aside. (PNTL, Interview 1)

They also noted an untapped potential for cooperation with PCIC so that a greater range of forensic disciplines could be used as needed:

Like we’re going to compare the equipment we have. Maybe we can learn from them; they can learn from us. (PNTL, Interview 8)

There seemed to be a need for support to facilitate collaboration within and between agencies.

### 3.5 Future vision

Participants were grateful for what had been received, and they had ideas and a vision with some sense of urgency, about what more was needed to maintain momentum and see real change.

#### 3.5.1 Appreciation for what has already been received

All PNTL participants expressed their appreciation for what had already been received and the quality of advisory support:

I don’t know how [to express] how lucky we are to have her here – as a specialist herself! You learn with the specialist – it’s different to learning with the tutor, right? So, if you were confused about something, she will tell you right away. Like fingerprint one, when you take a picture – she said, “Oh the ridges are not that strong – skip this one and move to another one – it’s strong”. This guidance really made us improve in selecting our target. (TLPDP, Interview 7)

So TLPDP creates a complete one. They start from the building and then provide the equipment, and they continue the training that they provide to us. They want to develop us …. they prepare us …. Because TLPDP never leaves us …, they are always together with us and whatever we do, they always accompany us and if there is any limitation that we have, they always together with our Commander, look at it, and if language is a barrier they try to fix it with the training, and if they see space is not enough for us, they identify this together with the Commander and look at this. (PNTL, Interview 3)

#### 3.5.2 Need for ongoing training and support

Participants expressed the hope that the TLPDP would continue to support them:

And what I hope for the future is still people will stay with us …. and our pace of developing. So, I will hope TLPDP stays with us and guides us every year. (TLPDP, Interview 7)

When asked about specific support needs, most members deferred to the leadership and hierarchy. When pressed, however, they reported the need for more training and how it may equip them to support other police officers and the community:

For our forensic team, I think that if TLPDP continues to provide training to us and continues to support us, one day we will have the knowledge and can provide training to other officers …. And we want to continue to provide training to other districts[/municipalities] – a couple of them have happened and we want to continue for more. (PNTL, Interview 5)

We need more knowledge of the area of forensic so we can better the investigation and minimise the concerns of the public. (PNTL, Interview 1)

#### 3.5.3 New forensic capabilities

Participants from the PNTL hoped that additional forensic capabilities could become a reality in the way that they could see fingerprint examination developing. Digital forensics was identified as a relevant next step given the ubiquity of mobile phones and their use in a range of crime types. There was agreement about the relevance of digital forensics within the PNTL and TLPDP, and it is identified as a specific area in the TLPDP's next Program Design Document 2023–2028 [43]. Additionally, PNTL participants were keen to learn more about ballistics and DNA analysis:

Because in future we will have more problems, more organised crime and other crimes, so we need more training in that area. So now we already have fingerprint training but for the future, many other areas we need to be training. DNA, ballistics … so in the forensics, we need to continue training! (PNTL, Interview 2)

DNA analysis seemed particularly alluring. Current arrangements were to send samples to Portugal or sometimes to Australia (AFP laboratory in Canberra) for analysis (Notes from TLPDP, Informal Interview). Recognising that DNA specialists and a purpose-built laboratory were a long way off for Timor-Leste, and unsure that PCIC had capacity, there was growing interest in the opportunities afforded by Rapid DNA:

Maybe portable DNA to narrow down the list [of suspects] when a case happens and also, it’s going to prepare for the DVI, the disaster identification system. It’s already happened before – people die when flooding happens (PNTL, Interview 8)

Having recently purchased two Rapid DNA devices, the NTPF Forensic Science Branch was about to undertake work on validation. They advised that each sample would cost approximately $500 (Australian) to test (Notes from NTPF, Informal Interview). For Timor-Leste, there are not only questions of set-up and consumables, but also the collection and storage of DNA samples and profiles, and establishment of a database, not to mention legislative frameworks [9]. Given a small budget for the PNTL in general, and for the National Forensic Team specifically, even covering the costs of personal protective equipment was a challenge, with the TLPDP providing support during the pandemic [25]. Unfortunately, therefore, the desire to include Rapid DNA does not align with current or projected funding circumstances.

Although the Northern Territory's forensic laboratory in Darwin is much closer to Dili (a direct flight of approximately 1 h 20 min) than is the AFP's laboratory in Canberra, arrangements had not been made to maximise the opportunity that may afford [25]. This is because the AFP is the Australian police organisation that has an international remit. The NTPF Forensic Science Branch is a modern and well-equipped laboratory that offers crime scene examination, fingerprint examination, forensic chemistry (illicit drug analysis and toxicology), forensic biology and digital forensics. The NTPF Forensic Science Branch also has agreements for some forensic services not currently available within the Northern Territory and at times seeks interstate technical review in circumstances where only one expert is available (Notes from NTPF, Informal Interview, clarified via personal communication). In the spirit of exchange, the NTPF Forensic Science Branch was open to exploring ways to assist the AFP and Timor-Leste with DNA or other forensic analysis.

## 4 Discussion

This aim of this study was to explore forensic capacity development in Timor-Leste from a critical forensic studies perspective that aims to understand how forensic science is socially constructed in particular contexts [7]. In this case, the political and organisational arrangements include the bilateral partnership between the Governments of Timor-Leste and Australia for the Timor-Leste Police Development Program (TLPDP), a partnership of two decades to date [45]. The findings of the present study highlighted the value of long-term partnerships and ongoing support in the key achievements of the TLPDP and PNTL. These include the establishment of a functional laboratory, the development of skills in crime scene photography, the introduction of all stages of fingerprint examination from crime scene to court, and knowledge-sharing with forensic focal points based in the municipalities. These core achievements – along with practice in forensic anthropology, and increased English-language skills – provided a tangible uplift in team members’ confidence, generated momentum in the team, and garnered interest from leaders. Several findings warrant further discussion from a critical forensic studies perspective.

### 4.1 Forensic science as a social process

A key concern from a critical forensic studies perspective is that forensic science be recognised as a social process that involves human decision making [7]. Forensic science involves the risk of human error at each stage of the process [52,53]. Our findings suggest that participants experienced forensic science as a social process, perhaps because their access to the knowledge and use of forensic science relied on the training and equipment provided by the TLPDP. The specific interpersonal relationships involved in *doing* forensic science – for example, in opportunities for practice in forensic anthropology available because of the forensic advisor’s particular expertise – may place the social nature of the endeavour at the forefront of practitioners’ minds. Participants did not appear to overvalue the science in the sense of believing it to be infallible; rather, they showed curiosity and wanted to learn more to provide robust evidence to contribute to the PNTL, the community, and Timor-Leste. It may be valuable for the AFP to explore opportunities with the NTPF to further enhance PNTL forensic skills development [25]. This may be appropriate due to the openness of NTPF to explore the possibility, the opportunity for wider forensic exposure, and the proximity, similar climate, and shared organisational focus on regional and remote policing. Support could take the form of laboratory visits and internships as well as examination when needed in forensic disciplines that are not yet available in Timor-Leste.

### 4.2 Forensic science within particular contexts

Another key concern of critical forensic studies is to understand that forensic science is constructed within particular economic, political, and cultural contexts [7]. Our findings reveal a great deal of complexity in the local environment given legacies of the past. They also highlight perceived support from leaders and the potential for greater collaboration between PCIC and PNTL. Such collaboration will introduce new challenges, but also the potential to enhance capacity through new team members and skills. Given Timor-Leste's circumstances, forensic science is necessarily developed in a partnership working towards the UN SDGs. For many TLPDP and PNTL members involved, there was no time to waste in developing forensic capacity, yet many elements needed to align to continue to make visible and tangible progress. The design document for the fifth iteration of the TLPDP from 2023 to 2028 [43] provides a plan for next steps in forensic capacity development, explicitly including the continuation of training and development in crime scene examination, crime scene photography, and fingerprint examination, along with data recording in a centralised system. Additionally, it aims to develop basic digital forensic capability through software upgrades and training and introduce in-field drug detection training and kits [43]. The plan is justifiable in that it involves strengthening the fledgling skills in fingerprint examination and exciting because it involves the introduction of new skills to maintain momentum.

However, among the key issues for forensic science capacity development are the sustainability of forensic science, and efforts towards meeting international standards [3]. The evaluation of TLPDP by Wilson et al. [25] noted that expenditure on forensic equipment to establish the PNTL forensic laboratory poses a sustainability risk. Here, it is important to recognise that increased capacity in a democratic police organisation is also beneficial for Australia and the region [15,17]. Further, this forensic capacity development has the potential to contribute to more robust evidence to guide police and legal decision-making, contributing to UN SDGs, and as participants in this study emphasised, a more effective organisation, and a safer country. If fingerprinting can be implemented successfully from crime scene to court, it will pave the way for the incorporation of further forensic disciplines and techniques. The AFP [43] noted that sustainability concerns are offset to some extent through improved standard operating procedures and safety, efficiency, and effectiveness. Supporting this potential, our findings suggest a sense of optimism among participants that leaders had listened to the rationale for human resource considerations in the forensic team. If the organisation supports the sustainability of forensic capacity through human resources initiatives, such as retaining forensic members and engaging in career and succession planning, forensic science is more likely to be sustainable. Additionally, the sustainability of fingerprint examination is supported by innovative approaches that use locally available consumables [50], which help to address high costs and supply disruptions [4,34].

### 4.3 Forensic science as contested knowledge

Although forensic science tends to have a privileged place in knowledge hierarchies [7], this place cannot be assumed in the Global South. Research on forensic capacity development in Ghana highlighted that one important milestone may be the availability of examples of the successful use of forensic science – whether as evidence to convict or as disconfirming evidence [3]. The publicity generated by such cases may help in raising awareness of forensic science among criminal justice practitioners and members of the public. Our findings in Timor-Leste highlight concerns among forensic team members that criminal justice practitioners – such as police investigators and lawyers – did not yet recognise the value of forensic science in relation to other forms of evidence, leading to a risk that it would be underused in criminal cases. Efforts will be needed to inform not only criminal justice practitioners, but also the public more broadly, about forensic science as forensic disciplines begin to be used by police, lawyers and judges to inform their decision-making. These concerns reflect similar issues in other jurisdictions of the Global South [3,5,8]. Further, even in non-adversarial systems, the negative impacts of flawed use or miscommunication of forensic science are most likely to be experienced by members of marginalised groups who are already overrepresented in criminal justice statistics. Given these inherent power relations, it is crucial that the forensic disciplines in use are understood by police, lawyers, judges, and the community at large. These issues highlight the need for appropriate communication between criminal justice actors to achieve just outcomes, as has been found in research in the Global North [7,[53], [54], [55], [56], [57]].

## 5 Limitations and future research

This study aimed to integrate findings from site visits, observations, and insights from formal and informal interviews to explore forensic capacity development in Timor-Leste. We were able to obtain rich data on the forensic capacity development partnership project. However, we focused narrowly on the National Forensic Team of the PNTL and the TLPDP, rather than undertaking a broader study including PNTL members involved in crime scene examination in the municipalities, the PNTL investigations team, and wider PNTL leadership. Findings from the present study suggest the need for forensic literacy training for all police officers as well as prosecutors and other legal practitioners. It was beyond the scope of the study to explore the quality and availability of science education in Timor-Leste. Research on science education would be helpful not only to understand the pathways for prospective forensic scientists but also to inform approaches to forensic literacy for criminal justice practitioners and the public.

The findings also suggest a need to initiate dialogue with other criminal justice practitioners [[54], [55], [56]], about forensic capacity in Timor-Leste. Notably, this includes PCIC members, particularly as the PCIC and PNTL merge. Further research could include a range of practitioners from first responders through to courts to gain insights from their perspectives regarding any potential issues in the flow of forensic science through the criminal justice system [53]. For example, exploring health, medical, and legal practitioners’ experiences with the use of forensic medical protocols as evidence would be valuable, especially given that forensic medical examinations are now available not only in the capital, but also in selected municipal hubs with a plan for further expansion [12]. It would also be helpful to examine forensic-related standard operating procedures and legislation, noting that a need has been found in other countries of the Global South [3,58] and observed by practitioners involved in capacity development [5]. Finally, while this study focused on Timor-Leste, further social sciences research is needed on forensic capacity development in a range of countries and contexts to better understand differences and local nuances, overlaps in challenges and solutions, and the role of local knowledge in contributing to effective partnerships and sustainable forensic science.

## 6 Conclusion

Forensic capacity development often occurs as a small and specialised component of broader police capacity development programs. Given the need for specialist practitioners and the costs associated with infrastructure, instruments, and consumables, the development of forensic capacity poses many challenges for countries of the Global South. This study aimed to explore forensic capacity development as part of the Timor-Leste Police Development Program, which reflects United Nations SDG 17 as a partnership for the goals between Timor-Leste and Australia. Adopting a critical forensic studies approach, the study prioritised a contextual understanding of how forensic science is socially constructed in Timor-Leste through the partnership. It found that although the partnership generated optimism, was regarded as productive, and valuable achievements had been made, there was a sense of urgency about next steps and the need for ongoing momentum. The findings also suggest that the achievements at this stage are somewhat precarious due to the need for extended training and development, challenges in expediting broader training, and untapped potential in inter-agency collaboration. Overall, long-term organisational commitment and ongoing high-quality partnerships are essential to facilitate sustainable forensic capacity development.

## CRediT authorship contribution statement

**Loene M. Howes:** Writing – review & editing, Writing – original draft, Project administration, Methodology, Investigation, Formal analysis, Conceptualization. **Roberta Julian:** Writing – review & editing, Investigation, Funding acquisition, Conceptualization. **Renee Wilson:** Writing – review & editing, Resources, Project administration, Conceptualization. **Maria C. dos Santos:** Resources, Project administration, Conceptualization.

## Declaration of competing interest

The authors declare the following financial interests/personal relationships which may be considered as potential competing interests:

Renee Wilson is employed by the Australian Federal Police. Maria Dos Santos is employed by Polícia Nacional de Timor-Leste. Roberta Julian is an editorial board member of *Forensic Science International: Synergy*. The authors received a waiver on the article publishing charge for open access.

## Footnotes

1 Acronyms: AFP – Australian Federal Police; NTPF – Northern Territory Police Force; PCIC - Polícia Científica de Investigação Criminal (Criminal Investigation Scientific Police); PNTL – Polícia Nacional de Timor-Leste (National Police of Timor-Leste); TLPDP – Timor-Leste Police Development Program; SDGs – sustainable development goals.

2 The Northern Territory Police Force (NTPF) was previously under the organisational umbrella of the Northern Territory Police, Fire and Emergency Services (NTPFES). Legislation was passed in 2023 that would make NT Police a separate entity (https://pfes.nt.gov.au/tri-services-transition; accessed 196.08.2024). The NTPFES was in a transition phase at the time of writing. We have referred to the NTPF throughout the article.

3 https://www.interpol.int/en/Who-we-are/Member-countries/Asia-South-Pacific/TIMOR-LESTE (accessed 16.08.2024).
